# Longevity of Literary Women

**Published:** 1854-04

**Authors:** 


### LONGEVITY OF LITERARY WOMEN.

The following examples show that devotion to literary duties is not necessarily destructive to the health and lives of women:

| Name. | Died. | Age. | Name. | Died. | Age. |
|---|---|---|---|---|---|
| Mrs. Hofland | 1844 | 74 | Miss Birney | 1840 | 88 |
| Jane Porter | '50 | 74 | Hannah Moore | '33 | 88 |
| Mrs. Chapone | '01 | 75 | Joanna Bailey | '51 | 89 |
| Mrs. Sherwood | '51 | 77 | Mrs. Carter | '06 | 90 |
| B. Maria Roche | '45 | 80 | Jane West | '52 | 93 |
| Mrs. Barbauld | '25 | 82 | Hon. Mrs. Monkton | '40 | 94 |
| Mrs. Piozzi | '21 | 82 | Harriet Lee | '51 | 95 |
| Mrs. Edgeworth | '49 | 82 | Mrs. Garrick | — | 97 |
| Mrs. Amelia Opie | '53 | 85 | Caroline L. Herschell | '46 | 98 |

